# Identification of a potential non-coding RNA biomarker signature for amyotrophic lateral sclerosis

**DOI:** 10.1093/braincomms/fcaa053

**Published:** 2020-06-17

**Authors:** Greig Joilin, Elizabeth Gray, Alexander G Thompson, Yoana Bobeva, Kevin Talbot, Jochen Weishaupt, Albert Ludolph, Andrea Malaspina, P Nigel Leigh, Sarah F Newbury, Martin R Turner, Majid Hafezparast

**Affiliations:** f1 School of Life Sciences, University of Sussex, Falmer, Brighton, UK; f2 Nuffield Department of Clinical Neurosciences, University of Oxford, Oxford, UK; f3 Centre for Neuroscience and Trauma, Blizard Institute, Queen Mary University of London, London, UK; f4 Department of Neurology, University of Ulm, Ulm, Germany; f5 Department of Neuroscience, Brighton and Sussex Medical School, University of Sussex, Falmer, Brighton, UK; f6 Department of Clinical and Experimental Medicine, Brighton and Sussex Medical School, University of Sussex, Falmer, Brighton, UK

**Keywords:** amyotrophic lateral sclerosis, ALS, biomarker, non-coding RNA, RNA-seq

## Abstract

Objective biomarkers for the clinically heterogeneous adult-onset neurodegenerative disorder amyotrophic lateral sclerosis are crucial to facilitate assessing emerging therapeutics and improve the diagnostic pathway in what is a clinically heterogeneous syndrome. With non-coding RNA transcripts including microRNA, piwi-RNA and transfer RNA present in human biofluids, we sought to identify whether non-coding RNA in serum could be biomarkers for amyotrophic lateral sclerosis. Serum samples from our Oxford Study for Biomarkers in motor neurone disease/amyotrophic lateral sclerosis discovery cohort of amyotrophic lateral sclerosis patients (*n* = 48), disease mimics (*n* = 16) and age- and sex-matched healthy controls (*n* = 24) were profiled for non-coding RNA expression using RNA-sequencing, which showed a wide range of non-coding RNA to be dysregulated. We confirmed significant alterations with reverse transcription-quantitative PCR in the expression of hsa-miR-16-5p, hsa-miR-21-5p, hsa-miR-92a-3p, hsa-piR-33151, TRV-AAC4-1.1 and TRA-AGC6-1.1. Furthermore, hsa-miR-206, a previously identified amyotrophic lateral sclerosis biomarker, showed a binary-like pattern of expression in our samples. Using the expression of these non-coding RNA, we were able to discriminate amyotrophic lateral sclerosis samples from healthy controls in our discovery cohort using a random forest analysis with 93.7% accuracy with promise in predicting progression rate of patients. Importantly, cross-validation of this novel signature using a new geographically distinct cohort of samples from the United Kingdom and Germany with both amyotrophic lateral sclerosis and control samples (*n* = 156) yielded an accuracy of 73.9%. The high prediction accuracy of this non-coding RNA-based biomarker signature, even across heterogeneous cohorts, demonstrates the strength of our approach as a novel platform to identify and stratify amyotrophic lateral sclerosis patients.

## Teaser

A set of small non-coding RNA were found to be differentially expressed in the serum of patients with amyotrophic lateral sclerosis compared to healthy controls. Using their dysregulation in a random forest model, we could classify the samples’ disease state with 73.9% accuracy in our confirmation cohort.

## Graphical Abstract

**Figure:**
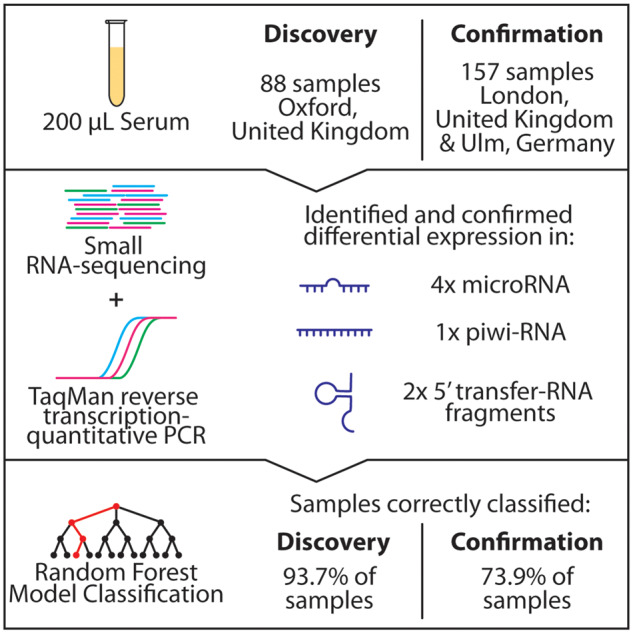

## Introduction

The majority of those with the adult-onset neurodegeneration amyotrophic lateral sclerosis (ALS) typically develop a syndrome of rapidly progressive muscle weakness secondary to motor neuronal loss, resulting in death within 3 years of first symptoms, typically from ventilatory insufficiency (Kiernan *et al.*, 2011). Despite a common clinical core of combined upper and lower motor neuron signs, there is variation in the mix of clinical signs, the site of initial weakness and the rate of disability progression, which contribute to a persistent diagnostic delay for many. The identification of biomarkers has been recognized as a priority for therapeutic development in a clinically heterogeneous syndrome like ALS, for determining subgroups in relation to pathogenesis and phenotype, and as early indicators of treatment response (Turner *et al.*, 2009). Biomarker sources that are minimally invasive, ubiquitously available and time efficient in their measurement are key and those derived from biofluids are well suited for this.

One class of biofluid-based molecules increasingly investigated as potential biomarkers are short non-coding RNA species (ncRNA). MicroRNA (miRNA) are common candidates as they have relatively simple structures, increased stability from RNase degradation and freeze-thaw cycles, and are present in blood (Chen *et al.*, 2008; Jin *et al.*, 2013). Dysregulated miRNA in the biofluids of ALS patients, including cerebrospinal fluid (CSF), and in blood-derived components plasma and serum, have been found, (summarized in Joilin *et al.*, 2019). While hsa-miR-206 has emerged as a leading candidate and shown previously up-regulated in ALS patient muscle biopsies (Bruneteau *et al.*, 2013; Russell *et al.*, 2013; Toivonen *et al.*, 2014; de Andrade *et al.*, 2016; Di Pietro *et al.*, 2017; Waller *et al.*, 2017*a*), this is not specific to ALS and few other miRNA candidates are common across studies. However, only a small subset of circulating ncRNA have been investigated. Several other ncRNA species are present in serum including ribosomal RNA (rRNA) and transfer RNA (tRNA) which have been used as potential biomarkers in other diseases (Liao *et al.*, 2010; Cheng *et al.*, 2014; Lopez *et al.*, 2015; Iliev *et al.*, 2016; Umu *et al.*, 2018; Yoffe *et al.*, 2018). Efficient investigation of these ncRNA requires the use of RNA-seq, and to date only one study has detected ncRNA in ALS biofluid samples, though they did not further consider these as biomarkers (Matamala *et al.*, 2018). We hypothesized that multiple ncRNAs are dysregulated in ALS, potentially reflecting key aspects of the syndrome’s clinical heterogeneity. Therefore, using minimal amounts of serum, we undertook an RNA-seq screen followed by TaqMan RT-qPCR to identify ncRNA-based diagnostic and prognostic biomarkers in ALS. We identified seven dysregulated ncRNA in the serum of ALS patients and showed integrating the expression of this set of ncRNAs using a random forest algorithm correctly categorize the samples with 93.7% and 73.9% accuracy in the discovery cohort and a distinct confirmation cohort of samples, respectively.

## Materials And Methods

### Patient information

The discovery cohort of samples were patients recruited as part of the Oxford Study for Biomarkers in MND/ALS (BioMOx) from the Oxford MND Centre. Ethical approval for extraction and use of the biofluid samples and associated clinical data were obtained from South Central Oxford Ethics Committee B (08/H0605/85) and NRES Central Committee South Central—Berkshire (14/SC/0083). All participants provided written consent (or gave permission for a carer to sign on their behalf). The study included patients with ALS, patients with ALS mimic conditions [including multifocal motor neuropathy and Kennedy’s disease; disease mimics (DM)] and healthy control (HC) subjects. Patients with ALS and mimic disorders were diagnosed according to standard criteria by two experienced neurologists (M.R.T., K.T.) and clinically re-assessed on the day of sampling (A.G.T., M.R.T.). For the confirmation cohort of samples, these were obtained from the ALS Biomarker Study from Queen Mary, University of London, and the Ulm Neurological Biobank from the University of Ulm to form the confirmation cohort. Participants from the ALS Biomarkers Study (London—City & East Research Ethics Committee 09/H0703/27) were consented in the study and baseline samples taken at or shortly after diagnosis by MND neurologist (A.M.), based on established criteria. This cohort of samples included healthy controls, disease mimics, neurological controls and ALS patients. Samples from the Ulm Neurological Biobank (Ethics Committee of Ulm University, proposal number 20/10, year 2010; *n* = 16) were admitted to the Department of Neurology at Ulm University Hospital, Germany and were diagnosed by an MND neurologist (A.L.), and included neurological controls and ALS patients. ALS disease mimics in the confirmation cohort were similar to the BioMOx cohort, while neurological controls included multiple sclerosis, migraine, amnesia and spinal ischaemia.

For both cohorts, healthy control subjects were typically spouses and friends. Additional clinical information was collected, including information relevant to this study like time from disease onset, diagnostic delay, mean disease duration, site of disease onset and progression rate to last visit, calculated as 48 (approximation of neurological status at disease onset minus ALSFRS-R score at the time of sampling divided by time from onset to sampling in months). For both cohorts, patients were classified as having fast- or slow-progressing ALS based on their rate of change in the ALSFRS-R clinical rating, with a threshold of 0.6 points per month selected based on the median value seen clinically to divide the samples (Table 1). The number of ALS cases on percutaneous endoscopic gastrostomy (PEG) or non-invasive ventilation (NIV) at the time of sampling are also reported. Lastly, genetic testing for the most common mutations were undertaken on the samples, though samples in The ALS Biomarker Study were only tested for C9orf72 expansions.

**Table 1:** Characteristics of the sample cohorts whose serum was used in this study

	Discovery cohort				Confirmation cohort							
	BioMOx				The ALS Biomarker Study					Ulm Neurological Biobank		
	HC	DM	ALS-SP	ALS-FP	HC	NC	DM	ALS-SP	ALS-FP	NC	ALS-SP	ALS-FP
Number of patients	24	16	24	24	46	23	27	22	23	10	5	1
Sex (M/F)	14/10	14/2	21/3	17/7	26/20	7/16	19/8	19/3	12/11	6/4	3/2	1/0
Age (years)	57.9 (34–72)	59.4 (35–82)	59.6 (46–76)	60.8 (31–81)	55.9 (40–71)	51.2 (27–90)	58.9 (21–79)	63.6 (43–75)	60.7 (40–82)	62.6 (40–73)	60.2 (41–70)	69
Progression rate	–	–	0.32 (0.05–0.6)	1.27 (0.59–5.07)	–	–	–	0.16 (0.01–0.59)	1.56 (0.70–4.41)	–	0.26 (0.04–0.58)	5.28
Mean disease duration (month)	–	–	30.8 (5.3–88.5)	14.1 (2.37–25.53)	–	–	–	93.7 (8.6–274.6)	17.2 (3.5–53.5)	–	38.25 (25–48)	9
Onset (spinal/ bulbar/both)	–	–	15/7/0	17/7/0	–	–	–	16/5/1	15/7/1	–	5/0/0	1/0/0
PEG/NIV	–	–	–	–	–	–	–	12	11	–	0	0
Genetic cases	–	–	C9orf72 (2) SOD1 (1)	C9orf72 (1)	–	–	–	1	6	–	C9orf72 (2) TARDBP (2) SOD1 (1)	SOD1 (1)
								Only screened for C9orf72				

PEG/NIMV information was not available for the ALS patient samples from the BioMOx discovery cohort. C9orf72 expansions were the only ALS-related mutations checked in The ALS Biomarker Study.

HC = healthy controls; NC = neurological controls; DM = disease mimics; ALS-SP = slow-progressing ALS patients; ALS-FP = fast-progressing ALS patients; M/F = male/female; PEG/NIV= percutaneous endoscopic gastrostomy/non-invasive ventilation.

### Sample collection, preparation and RNA extraction

Blood was collected from patients into BD Vacutainer SST tubes, left to clot at room temperature and centrifuged at 3000 rpm for 10 min at 4˚C. The serum supernatant was then removed and aliquoted into 1.8 ml aliquots and stored at −80˚C. Minimal red blood cell lysis was checked using a haemoglobin ELISA (ab157707, Abcam) with a threshold of 0.6 g/l (Lippi *et al.*, 2006), with one disease mimic sample excluded. Small RNA was isolated from a 200 µl sub-aliquot of individual serum samples using the miRNeasy Micro kit (Qiagen) with a DNase I treatment (Qiagen). Qubit miRNA assay (Invitrogen) quantification was used to give relative amounts of small RNA present in each sample.

### RNA sequencing

Healthy control, ALS-SP and ALS-FP samples from the BioMOx discovery cohort were split into three age- and sex-matched groups of eight samples each. For each pool, 2.5 ng of small RNA per sample were combined and concentrated using a SpeedVac at ambient temperature for 40 min from 50 µl to 8 µl. Each sample pool was then converted into RNA-Seq libraries using the QIAseq miRNA library kit (Qiagen) following the recommended parameters for serum RNA. These libraries then underwent 75 bp PE sequencing on an Illumina MiSeq machine at the Oxford Genomics Centre, United Kingdom.

Data were pre-processed to remove 5′ and 3′ adaptors and then underwent two analysis pipelines. The automated Qiagen QIASeq miRNA pipeline read the unique molecular indexes (UMI), sequentially aligned the RNA-seq data to a database of ncRNA transcripts using Bowtie. The second method used the Galaxy web servers using a range of open access script packages (Afgan *et al.*, 2018). Adapter only, short or low quality reads were removed using Trim, and reads were then aligned using Salmon (Patro *et al.*, 2017) to a database of ncRNA from miRBase, piRNA Bank (piRNA with NCBI accession numbers), gtRNAdb and Ensembl, with transcripts with the same sequence merged together. Reads from both Qiagen and Galaxy were then normalized and differential expression calculated between sample groups using DESeq2 (Love *et al.*, 2014).

### RT-qPCR confirmation and analysis

Using the TaqMan Advanced miRNA RT-qPCR chemistry, a fixed volume of 2 µl of RNA extracted from serum from both the BioMOx discovery cohort and The ALS Biomarker Study and Ulm Neurological Biobank confirmation cohort was converted to cDNA with pre-amplification and diluted 10-fold. Three samples from the healthy controls and fast-progressing ALS patients were not included because of insufficient RNA. Using pre-designed primers and master mixes (Applied Biosystems; miRNA commercially available probes: Supplementary Table 1; piRNA and tRNA: custom-designed probes), candidate transcripts were quantified using fast cycling conditions on an ABI Viia7 cycling machine. Cq values were averaged and normalized to the arithmetic average of hsa-miR-718 and hsa-piR-31068 (DQ570956), which were identified using NormFinder (Andersen *et al.*, 2004). An average for the healthy control samples was calculated and all samples were compared to this average for a ΔΔCq value.

### Statistical analysis and modelling

Statistical analysis for RT-qPCR were conducted on ΔΔCq values for each sample with GraphPad Prism 6.0 and SPSS v24. Outliers were identified using the ROUT method in GraphPad Prism 6.0 (Q = 1%). Distribution of the data was determined using a Shapiro–Wilks normality test. One-way ANOVA was carried out across the four groups with Tukey’s multiple comparison for parametric data and a Welch’s one-way ANOVA with Gomes–Howell multiple comparison when non-parametric. Correlations were analysed using Pearson’s and Spearman’s rank correlation tests if the data were parametric and nonparametric, respectively. All statistics were two-tailed and significance was set at *P* < 0.05.

Modelling of the data to predict sample classification was carried out using Orange. Three BioMOx samples that underwent RT-qPCR expression profiling were excluded from this analysis as they did not have expression data across the six ncRNA biomarkers showing differential expression. Hsa-miR-206 was not included as it does not have a normally distributed expression pattern. Normalized ΔCq data for the remaining samples were run through a random forest model, generating 10 replicable trees with no subset smaller than 5. Disease state was the grouping variable, with the ncRNA as independent variables entered together.

### Data availability

The data that support the findings of this study are available from the corresponding author, upon reasonable request.

## Results

### Small RNA-seq analysis highlights dysregulated ncRNA in the serum samples from ALS patients

To obtain an overview of small ncRNA expression dysregulation in ALS, we profiled serum samples from slow- and fast-progressing ALS patients, and healthy controls from the BioMOx discovery cohort. Small ncRNA extracted from 200 µL of serum were pooled together, with libraries created from these pools to improve the signal-to-noise ratio as we have shown previously (Jones *et al.*, 2012; Caserta *et al.*, 2016; Caserta *et al.*, 2017). Using Illumina-based RNA-seq, 3,438,537 reads per pooled sample on average were generated, which we analysed using two different approaches: the Qiagen automated analysis (QA) and an optimized open-sourced software-based workflow hosted on the Galaxy web servers (GA).

**Figure 1:**
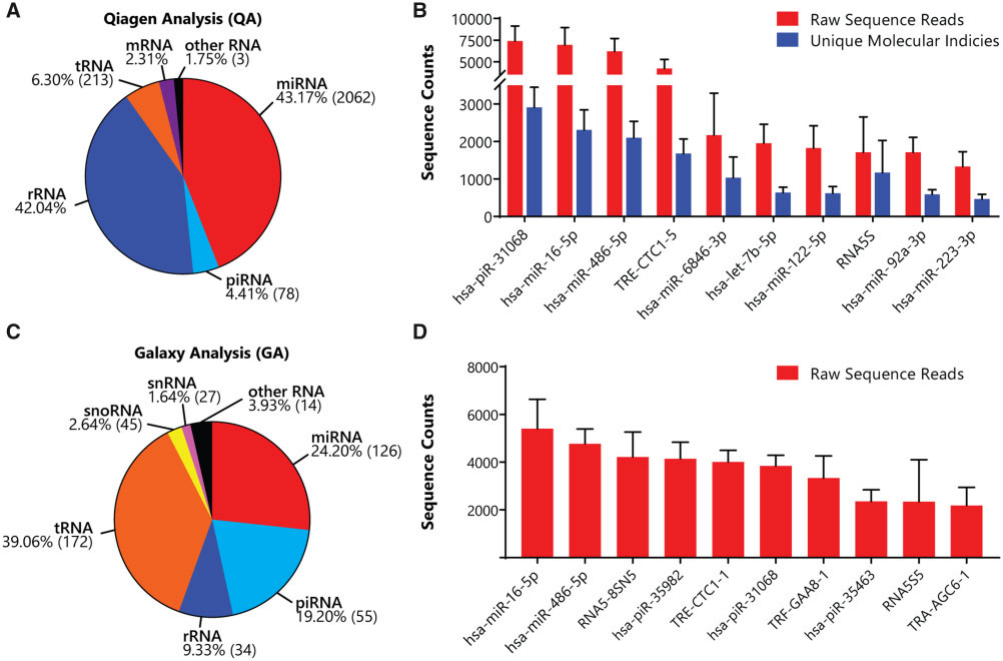
**Serum ncRNA reads for small RNA-seq.** (**A**) Proportion of total aligned reads annotated to ncRNA species across all sample pools for the Qiagen-based analysis (QA). Other ncRNA species includes hairpin RNA, snRNA, snoRNA and Y RNA. Number of distinct transcripts are in brackets, except for rRNA and mRNA, which QA does not provide. (**B**) Top ten read targets present on average across all samples, shown as raw sequencing reads, and after unique molecular indexes have been taken into account for QA. (**C**) Proportion of total aligned reads annotated to ncRNA species across all sample pools for the Galaxy-based analysis (GA). Other ncRNA species includes scRNA, sRNA, mt_rRNA, mt_tRNA, scaRNA and vaultRNA. Number of distinct transcripts are in brackets. (**D**) Top ten read targets present on average across all samples in GA, shown as raw sequencing reads. Bars: average ± SEM.

Following pre-processing, QA aligned 17.5% of reads to annotated sequences on the human genome (Supplementary Table 2), and although this is low, this is reflective of the variable intact status of RNA in serum and consistent with previous studies (Waller *et al.*, 2017b; Coenen-Stass *et al.*, 2018; Matamala *et al.*, 2018; Dufourd *et al.*, 2019; Wong *et al.*, 2019). Across all sample pools, most aligned reads were miRNA, followed by rRNA and tRNA, with none of the other ncRNA species including piRNA making more than 5% of all ncRNA (Fig. 1A;Supplementary Table 3). On average, seven of the top ten read targets present across all sample pools were miRNA, though the piRNA hsa-piR-31068 (NCBI: DQ570956) had the highest count (Fig. 1B), its high expression previously seen in serum from colon cancer patients (Vychytilova-Faltejskova *et al.*, 2018). Correction with the randomly generated hexamer unique molecular indexes (UMI) for clonal amplification bias during the polymerase chain reaction (PCR) amplification of the libraries made no difference to the overall proportion of ncRNA, supported by a strong positive Spearman’s correlation before and after [r_s_ (2369) = 0.9724, *P* < 0.001], and consistent with previous studies showing minimal clonal amplification bias (Jayaprakash *et al.*, 2011; Fuchs *et al.*, 2015). However, QA undertakes a sequential annotation approach biasing towards miRNA, as any reads that align to miRNA are then subsequently removed from the analysis. This may in part lead to the results being skewed if the read is from another ncRNA, thus reducing the ability to detect other ncRNA. Additionally, QA restricts differential expression calculations to miRNA and piRNA.

Therefore, we complemented our analysis by using an open-sourced software hosted on the Galaxy web servers (GA) for concurrent alignment with a custom-made annotation file of all ncRNA transcripts including miRNA, rRNA, tRNA, piRNA, small nucleolar RNA (snoRNA) and snRNA. As piRNA is annotated to multiple loci on the genome and may be discarded for multiple alignments, we used the transcript aligner Salmon which generated an average alignment of 6.4% to annotated sequences, allowing for multiple mapping due to the short nature of the reads and target sequences (Supplementary Table 3). While lower than QA, this reduction in alignment is likely due to not including longer rRNA sequences such as 18S, 28S and 45S in our annotations, as their inclusion increased alignment to 12% on average. Indeed, this is not surprising as our GA pipeline aligns the reads to a specific subset of small ncRNA transcripts, or transcriptome, and not the whole genome. As such, reads that may align to mRNA or long ncRNA will not be detected and included in the analysis. Across all sample pools in the GA analysis, the highest number of reads aligned to tRNA, followed by miRNA, piRNA and other ncRNA species making up less than 5% of all aligned ncRNA reads (Fig. 1C;Supplementary Table 3). Those most expressed according to GA included three piRNA, three tRNA, two miRNA and two rRNA (Fig. 1D), five of them consistent with QA. This is reinforced by a limited correlation in the number of reads aligned and annotated to the same ncRNA between QA and GA [r_s_ (546) = 0.2653, *P* < 0.001].

**Figure 2:**
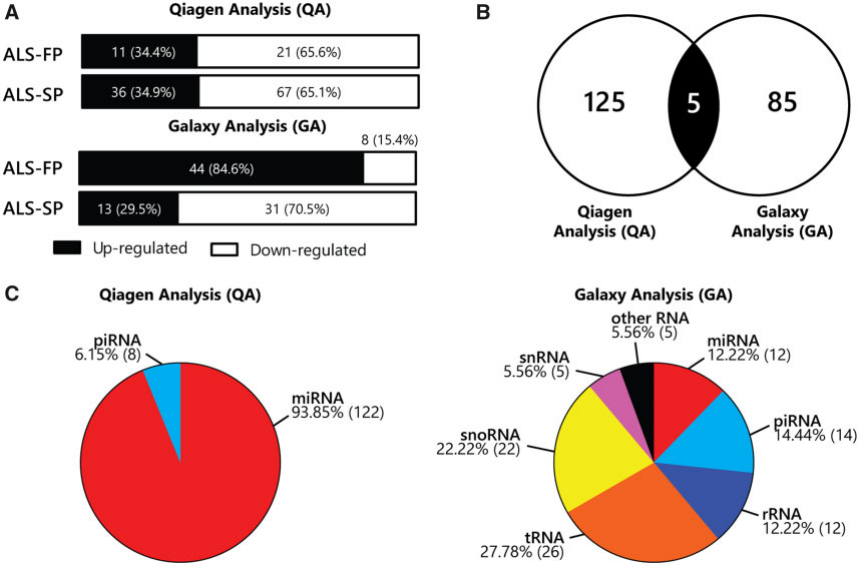
**Differential expression of ncRNA in the fast-progressing (ALS-FP) and slow-progressing (ALS-SP) ALS patients compared to healthy controls.** (**A**) Proportion of ncRNA reads differentially expressed (aligned reads > 5, *P* < 0.05) that are up-regulated or down-regulated across the two sample groups or the two analyses. (**B**) Overlap of ncRNA differentially expressed in both ALS groups between the two different analyses. Only five ncRNA were identified as being changed in both Qiagen-based analysis (QA) and Galaxy-based analysis (GA), with three being in the same direction in both. In addition levels of a further five and six ncRNA were altered in both ALS groups in QA and GA, respectively. However, the average expression of these ncRNA in the RNA-seq was low and thus were not included in those biomarkers assayed. (**C**) Proportion of ncRNA species detected as being differentially expressed in QA and GA. Number of distinct transcripts are in brackets.

These differences persisted into those ncRNA significantly dysregulated between ALS patients and healthy controls. QA showed 32 differentially expressed miRNA or piRNA in ALS-FP and 103 in ALS-SP (aligned reads > 5 per ncRNA across all samples, *P* < 0.05) (Fig. 2A) with similar proportions of up- and down-regulated ncRNA in the two groups. In contrast, GA, which identified 52 and 44 differentially expressed ncRNA in ALS-FP and ALS-SP respectively, showed non-consistent directional proportions (Fig. 2B). Further, differentially expressed ncRNA from QA and GA revealed minimal overlap (Fig. 2B), with only five targets overlapping and of these, three were dysregulated in the same direction [hsa-miR-9-5p, hsa-miR-142-5p and hsa-piR-33151 (DQ593039)]. This is despite a significant moderate correlation between the two analyses on all ncRNA and differential expression between the two groups [r_s_(489) = 0.3961, *P* < 0.0001; Supplementary Fig. 1A]. This is not surprising as not only are there large and significant differences in the ncRNA annotations and those included for differential expression affecting the normalization and the consequent results, in addition there are a low number of reads. To ensure there were no issues with the analysis methods, we undertook these two pipelines with an RNA-seq dataset from 200 ng of RNA from CD8+ T-cells (Heinicke *et al.*, 2020). While there was an increased alignment rate, there was a similar correlation of differentially expressed ncRNA between the two analyses [rs(1803) = 0.3934, *P* < 0.0001; Supplementary Fig. 1B]. Further, while there was a greater overlap of those trending in the same direction (91/105; 86%), this might be reflective of more robust changes between samples.

Of those significantly differentially expressed, QA showed miRNA were the dominant dysregulated species when compared only to piRNA (Fig. 2C). However, with GA detecting a wider range of ncRNA species, tRNA were the dominant species and consistent with their prevalence in reads overall (Figs 1C and 2C). Most species showed a decreased share of those differentially expressed in GA, largely driven by snoRNA making up a larger proportion of dysregulated ncRNA though these snoRNA were present just above the thresholds defined for differential expression (Fig. 2C).

### RT-qPCR confirms differential expression of six ncRNA across sample groups

To confirm the dysregulation of the most promising ncRNA identified in our pooled RNA-seq analyses, we used RT-qPCR on individual samples from the BioMOx discovery cohort. Using NormFinder on our RNA-seq data, we identified two stably expressed normalizers, hsa-miR-718 and the highly expressed piRNA, hsa-piR-31068. We confirmed this using RT-qPCR analysis on individual samples from the BioMOx discovery cohort, with an average Cq value of 28.2, a coefficient of variations of 7.2% and 8.7% across all samples respectively, with their average 7.4%, and their expression correlated with each other [r_s_ (81) = 0.7404, *P* < 0.0001].

**Figure 3:**
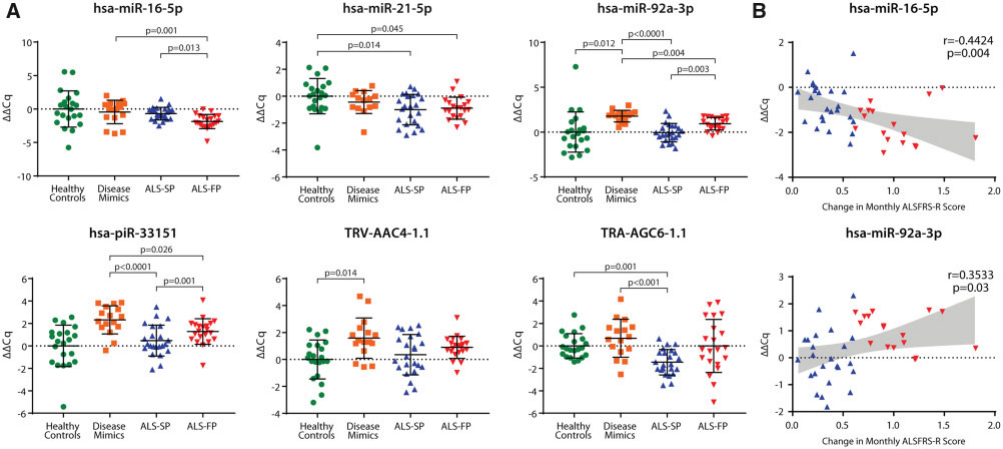
**Differential expression of ncRNA biomarkers in ALS patient serum samples using RT-qPCR in the BioMOx discovery cohort.** (**A**) Overall effects of disease state were found across all six ncRNA. (**B**) A correlation (Pearson’s) was found between the progression of ALS as determined by the monthly change in the ALSFRS-R score and hsa-miR-16-5p and hsa-miR-92a-3p expression. hsa-miR-21-5p/hsa-piR-33151: one-way ANOVA with Tukey post-hoc; hsa-miR-16-5p/hsa-miR-92a-3p/TRV-AAC4-1.1/TRA-AGC6-1.1: Welch’s one-way ANOVA with Games–Howell *post hoc*. Normalized to hsa-miR-718 and hsa-piR-31068. Relative expression to the average expression of healthy controls. Healthy controls *n* = 21, disease mimics *n* = 16, slow-progressing ALS (ALS-SP) *n* = 23, fast-progressing ALS (ALS-FP) *n* = 21. Bars: average ± SD.

We selected 20 ncRNA to assay from both the Qiagen- and Galaxy-generated lists independently, based on their differential expression, expression level and in the case of miRNA evolutionary conservation to ensure they reflect real differences. By using both lists, we were able to identify the best targets from both analyses since there was very little overlap and the analysis pipeline differed. Furthermore, potential biomarkers were initially selected from those that were differentially expressed in ALS-FP compared to controls, as the assumption was that any changes should be more pronounced in this group. Using TaqMan Advanced miRNA RT-qPCR assays, hsa-miR-16-5p, hsa-miR-21-5p, hsa-miR-92a-3p, hsa-piR-33151, TRV-AAC4-1.1 and TRA-AGC6-1.1 were confirmed to be differentially expressed between the four sample groups (Fig. 3A; fold changes presented in Supplementary Table 4). Significant dysregulation was observed between healthy controls and both ALS groups for hsa-miR-21-5p. Hsa-piR-33151, one of three differentially expressed ncRNA in both QA and GA, did not show dysregulation when compared to the healthy controls, but did with the ALS groups and to the disease mimics, the latter of which showed a greater but non-significant change compared to healthy controls. Detection of two 5′-tRNA fragments, TRV-AAC4-1.1 and TRA-AGC6-1.1, identified only a significant difference between the healthy controls and the disease mimics for the former, with the latter showing differences between ALS-SP and the healthy controls and disease mimics; a unique and unexpected expression pattern not seen in any of the other results.

While hsa-miR-16-5p showed no significant differential expression between the ALS patients and healthy controls, inconsistent with the RNA-seq, the differences between the disease mimics and both ALS-FP and ALS-SP was consistent with the RNA-seq results. Additionally, hsa-miR-92a-3p also showed no statistically significant differential expression between the ALS patients and healthy controls; however, it did show changes between the other groups. Interestingly, both these miRNA showed smaller variation in the ALS groups compared to the healthy controls, suggesting a previously observed disease-driven change in RNA expression (Ho *et al.*, 2008; Mar *et al.*, 2011). Further, only decreases in hsa-miR-16-5p and increases in hsa-miR-92a-3p serum expression were correlated with higher rates of disability progression measured by an average monthly change in ALSFRS-R score [hsa-miR-16-5p: r (41) = −0.4424, *P* = 0.004; hsa-miR-92a-3p: r (41) = 0.3533, *P* = 0.02; Fig. 3B). While both miRNA are enriched in red blood cells (Pritchard *et al.*, 2012), no significant correlation between their expression and haemoglobin levels was present [hsa-miR-16: r_s_ (79) = −0.1448, *P* = 0.20; hsa-miR-92-3: r_s_ (79) = 0.1972, *P* = 0.08), suggesting the changes did not reflect sample contamination. Comparison of biomarkers with age at time of sampling showed no significant correlation to the ncRNA expression. Hsa-let-7a-5p, hsa-let-7f-5p, hsa-miR-143-3p, hsa-miR-151a-3p, hsa-miR-451a, hsa-piR-36243 (DQ598177), hsa-piR-35982 (DQ597916), TRC-GCA2-2.1 and TRK-TTT3-1.1 were all tested but did not show significant dysregulation in ALS samples compared to controls (*P* > 0.05). Furthermore, hsa-miR-9-5p and hsa-miR-142-5p were shown to be differentially expressed in both RNA-seq analyses, but could not be confirmed with RT-qPCR. We also attempted to profile hsa-miR-493-3p and hsa-miR-6748-5p but were unable to get reliable amplicon amplification across all samples.

### Cross-validation of model and differential expression in a distinct cohort

**Figure 4:**
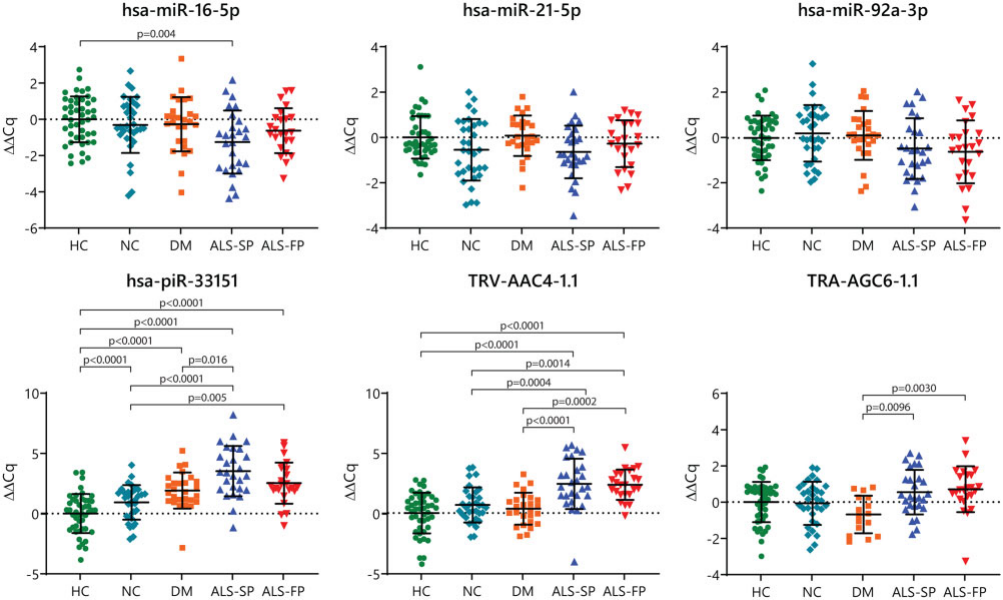
**Differential expression of ncRNA biomarkers in ALS patient serum samples using RT-qPCR in the ALS Biomarker Study & Ulm Neurological Biobank confirmation cohort.** Hsa-miR-16-5p/hsa-miR-21-5p/hsa-miR-92a-3p/TRV-AAC4-1.1/TRA-AGC6-1.1: one-way ANOVA with Tukey post-hoc, hsa-piR-33151: One-way ANOVA with Gomes–Howell *post hoc*. Normalized to hsa-miR-718 and hsa-piR-31068. Relative expression to the average expression of healthy controls. Healthy control (HC): *n* = 46; neurological controls (NC): *n* = 33; disease mimics (DM): *n* = 27; slow-progressing ALS (ALS-SP): *n* = 27; fast-progressing ALS (ALS-FP): *n* = 24. Bars: average ± SD.

To cross-validate our biomarkers identified from the BioMOx cohort, we obtained samples from The ALS Biomarker Study at Queen Mary, University of London, United Kingdom (*n* = 141) and the Ulm Neurological Biobank at the University of Ulm, Germany (*n* = 16). These samples in this confirmation cohort consisted of both slow- and fast-progressing ALS samples, along with healthy controls, neurological controls (including headaches, amnesia and multiple sclerosis) and disease mimics (neurological diseases similar to ALS). Firstly, with the addition of these samples, our normalizers continued to correlate with each other [r_s_ (156) = 0.7873, *P* < 0.0001] with a coefficient of variation of 7.8% and 9.3% for hsa-miR-718 and hsa-piR-31068, respectively. Using RT-qPCR, we profiled the expression of the six ncRNA that showed differential expression in the discovery cohort on these new samples, and found that while there was a trend for consistent regulation, these did not reach significance (Fig. 4). Indeed, dysregulation of hsa-miR-21-5p and hsa-miR-92a-3p was no longer detected in the confirmation cohort. In contrast, some of the biomarkers showed greater differences in the confirmation cohort with hsa-piR-33151 and TRV-AAC4-1.1 showing differences between the control and the ALS groups. Additionally, it can be noted that the small amount of variation detected in the ALS groups for ALS-SP and ALS-FP for hsa-miR-16-5p and hsa-miR-92a-3p in the BioMOx cohort was not seen in the new cohort, in which increased variation was seen across all biomarkers and sample groups. Additionally, no significant correlations between our six biomarkers and progression speed or haemolysis of our samples were identified.

While these results suggest that there are differences between our discovery and confirmation cohorts, we did group the two cohorts together to observe if there was an overall effect in the dysregulation of these ncRNA. This analysis showed that there was differential expression detected between our healthy controls and the ALS groups for a number of our biomarkers including hsa-miR-16-5p, hsa-piR-33151 and TRV-AAC4-1.1 (Supplementary Fig. 2). However, TRA-AGC6-1.1 showed no significant differential expression across any of the five groups.

### Hsa-miR-206 is detected mainly in disease samples but not healthy controls

**Figure 5:**
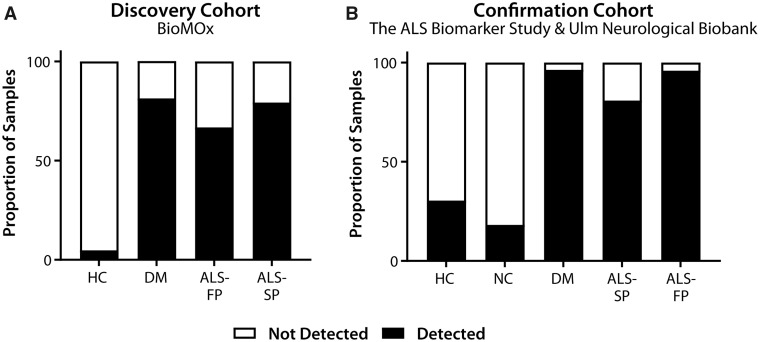
**Expression of hsa-miR-206 in our serum samples.** (**A**) Detection of hsa-miR-206 in the four sample groups in the BioMOx discovery cohort, showing a single sample with hsa-miR-206 expression in healthy controls, but in over 65% of the other three groups. (**B**) Detection of hsa-miR-206 in the combined confirmation cohort of samples from The ALS Biomarker Study & Ulm Neurological Biobank. A low number of samples in healthy and neurological controls expressed hsa-miR-206 in serum compared to the disease samples. BioMOx: Healthy controls (HC) *n* = 21, disease mimics (DM) *n* = 16, slow-progressing ALS (ALS-SP) *n* = 23, fast-progressing ALS (ALS-FP) *n* = 21; The ALS Biomarker Study & Ulm Neurological Biobank: HC: *n* = 46; neurological controls (NC): *n* = 33; DM: *n* = 27; ALS-SP: *n* = 27; ALS-FP: *n* = 24.

Both of our Qiagen and Galaxy analyses showed that hsa-miR-206 is upregulated in both ALS groups, but also in disease mimics, suggesting that this dysregulation of hsa-miR-206 is not specific to ALS. TaqMan RT-qPCR confirmation showed a unique pattern where hsa-miR-206 was only detected in one of 21 healthy control samples (4.8%) compared to the ALS-FP (66.7%) and ALS-SP (79.2%) samples, and disease mimics (81.3%; Fig. 5A) from the BioMOx discovery cohort. The RNA-seq data also highlighted hsa-miR-16-3p and hsa-miR-23a-5p as showing binary pattern of expression between the groups but RT-qPCR revealed little or no amplification of these miRNA across all samples. When hsa-miR-206 was tested in our confirmation cohort, we found a similar pattern of expression, with a low number of samples in our healthy controls (30.4%) and neurological controls (18.2%) expressing hsa-miR-206. Meanwhile, a high proportion of the disease mimic (96.3%) and ALS samples showed expression for hsa-miR-206 (ALS-SP: 80.8%; ALS-FP: 95.8%; Fig. 5B), suggesting that binary-like expression is consistent across cohorts.

### Random forest model accurately predicts sample classification

With any biomarker discovery study, it is important to determine if the targets identified are able to help assign samples to their specific diagnostic criteria. Therefore, we compared the sensitivity and specificity of the seven ncRNA biomarkers to each other to determine their ability to distinguish control samples (including the disease mimics) to that of the ALS patients in the BioMOx discovery cohort. Hsa-miR-16-5p (AUC = 0.7033), hsa-miR-21-5p (AUC = 0.7071), hsa-miR-92a-3p (AUC = 0.6429), hsa-miR-206 (AUC = 0.6864) and TRA-AGC6-1.1 (AUC = 0.7015) all showed a significant ability to distinguish the two groups apart. Combining the expression of the seven biomarkers in a logistic regression model however did not provide improved discrimination (AUC = 0.6162).

**Figure 6:**
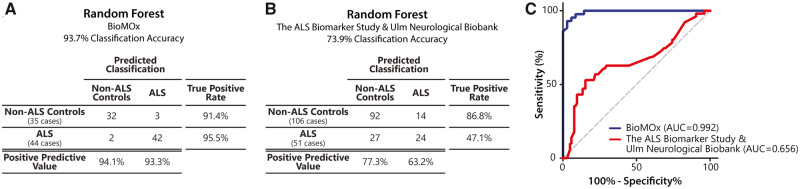
**Predictive classification modelling using the biomarkers and cross-validation.** (**A**) Confusion matrix of the sample classification from the random forest model generated from the BioMOx samples. Overall, 93.7% of all the samples entered into the model were correctly predicted to their observed classification. (**B**) Confusion matrix of the sample classification cohort on The ALS Biomarker Study and Ulm Neurological Biobank samples from the random forest model generated from the BioMOx cohort. Overall, 73.9% of all the validation samples entered into the pre-existing model were correctly predicted to their observed classification. (**C**) ROC graph of the random forest model showing the curves for the classifications of ALS patients in the BioMOx cohort (blue line; AUC = 0.9959) and in the cross-validation cohort from The ALS Biomarker Study and Ulm Neurological Biobank (red line; AUC = 0.656). Non-ALS controls include healthy controls, neurological controls, and disease mimics. ALS samples included both slow- and fast-progressing ALS patient samples.

To determine whether this could be improved, we investigated using predictive classification modelling to increase the predictive performance of our biomarker signature. We excluded hsa-miR-206 from the modelling as many require the data to be normally distributed, which the binary nature of hsa-miR-206 expression does not demonstrate. We used a random forest model, which creates a number of decision trees, and uses a majority vote approach to determine the classification of the sample. This random forest model classified our samples between ALS and non-ALS samples 93.7% of the time in our discovery cohort (AUC = 0.992; Fig. 6A and C). To test for overfitting, partitioning was undertaken, and resulted in continued high classification (100-fold two-third partitioning: CA = 73.9%, AUC = 0.785).

However, as the model was tested with the samples that underpinned it, we then utilized our samples from the confirmation cohort to test it. Interestingly, despite the differences in differential expression in our selected biomarkers in the confirmation cohort as described above, when we used the existing random forest model generated from the BioMOx samples to classify these new samples, we found that the model classified them with an accuracy of 73.9% (AUC = 0.656; Fig. 6B and C). One observation though was that ALS cases from the confirmation cohort were not as well classified as controls based on the BioMOx discovery cohort, most likely underpinned by the differences in expression between the two for some of our biomarkers. To explore this, we did undertake an analysis to partition the combined dataset (*n* = 236), using 20% of all the samples to create the model and to test the classification with the other 80%. Undertaking this analysis, we found that after an initial high classification (CA = 97.9%; AUC = 0.996), in the partition cross-validation, we continued to see an accuracy of 69.4% (AUC = 0.755). While this was less overall than the previous model, we did find there was a minor increase in the number of ALS samples that were classified in comparison (59.6%). Therefore, through using the expression of these biomarkers, this is a first step in establishing a biomarker signature to identify patients with ALS.

## Discussion

RNA-seq analysis enabled us to identify and confirm, using RT-qPCR, dysregulation of four miRNA, one piRNA and two tRNA across our sample groups. Together, this ncRNA biomarker signature can categorize over 90% of individual samples into whether they come from ALS and non-ALS patients in our discovery cohort and over 73% in a distinctly separate cohort of samples. As such, this shows the strength of using next generation RNA-seq to look at small ncRNA, but it is important to note that there are challenges at investigating ncRNA in the serum, chief among them are those caused by the low abundance of intact small RNA and increased levels of degraded longer RNA transcripts. While not new, they continue to be an issue as these degraded reads are able to soak up reagents and reads, which in part could result in low alignment scores we observed. This has been observed in other studies using the same RNA-seq kit and target tissue, including a study that also used 200 µl serum to find an average alignment of around 18% (Coenen-Stass *et al.*, 2018; Dufourd *et al.*, 2019; Wong *et al.*, 2019; Heinicke *et al.*, 2020). As such, this can limit the power of the analysis and may result in small but consistent dysregulation of those transcripts with low expression being missed, but these types of changes would not be suitable for use as a biomarker. Further, combined with the different approaches to what reads were aligned to (genome versus ncRNA transcriptome), this could explain why we saw small overlap in the targets identified between analyses. Therefore, careful consideration of the data in order to encompass the greatest number of possible targets must be undertaken, and so investigating all small ncRNA, not just miRNA, should be undertaken. As such, this is why we utilized the generated list of potential targets from both QA and GA since they both looked at different subsets of the ncRNA transcriptome through different methods.

Indeed, our RNA-seq identified non-miRNA ncRNA dysregulated in ALS patients. While the precise function of piRNA outside of gene regulation in germline cells is uncertain, their role in other tissues is starting to become evident. Indeed, for our ncRNA biomarker hsa-piR-33151, one of our most consistently changed biomarker in both cohorts, it has been reported to increase in the exponentially growing breast cancer cell line MCF-7 compared to those that are quiescent (Hashim *et al.*, 2014). We also detected the presence of two 5′-tRNA fragments (Park and Kim, 2018). While only one of these continued to show changes in our confirmation cohort, the presence of these fragments has been found to be induced by stress, and consequently affects RNA translation within U2OS cells and the assembly of stress granules (Yamasaki *et al.*, 2009; Emara *et al.*, 2010). It is possible that this might be occurring in ALS, where stress-related responses have been observed. Further work is required to investigate the sources and functions of these circulating ncRNA in ALS. hsa-miR-206, a myomiR or miRNA highly expressed in muscle, has previously been linked to ALS both biologically and as a biomarker (Williams *et al.*, 2009; Bruneteau *et al.*, 2013; Russell *et al.*, 2013; Toivonen *et al.*, 2014; de Andrade *et al.*, 2016; Di Pietro *et al.*, 2017; Waller *et al.*, 2017*a*). The working hypothesis is that hsa-miR-206 is released into the blood stream as a result of muscle death. As such, it is not surprising that in our disease control population, which is made up of neuropathies and other muscle related diseases, hsa-miR-206 is still detected—consistent with other studies identifying hsa-miR-206 as a blood-based biomarker for other muscle-related diseases (Matsuzaka *et al.*, 2014; Coenen-Stass *et al.*, 2017; Wang *et al.*, 2017). However, in our neurological control cohort, which has patients with headaches and amnesia, and are not motor related, very few patients show hsa-miR-206. Interestingly, while previous studies detected hsa-miR-206 in control samples, we were only able to detect it in disease samples in a binary-like expression pattern in our cohorts. We hypothesize this is likely due to the lower volume of serum that we used in comparison to other studies, and that hsa-miR-206 in healthy controls and neurological controls is so low that there is an insufficient amount to detect it even with RT-qPCR.

Importantly, the expression of none of our biomarkers were shown to correlate with the levels of haemolysis present in our samples, including hsa-miR-16-5p and hsa-miR-92a-3p which have been shown to be enriched in red blood cells (Pritchard *et al.*, 2012). While this suggests their expression is not due to their enrichment in red blood cells, we do recognize if this moves forward into a clinical setting that there is a risk that significant levels of haemolysis in patient samples could affect their expression, and affect their utility as part of the biomarker signature. Furthermore, while significant but weak correlations were found between monthly changes in ALSFRS-R score and the normalized Cq values of both hsa-miR-16-5p and hsa-miR-92a-3p in the discovery ALS samples, these did not translate through to the confirmation cohort. This may explain why the four group random forest model, which aimed to separate ALS patients based on progression speed, did not validate in the confirmation cohort. Interestingly, both of these miRNA have previously been reported to be down-regulated in the CSF of ALS patients using RNA-seq (Waller *et al.*, 2017*b*), suggesting there might be related regulation of these miRNA between these two biofluids. Paradoxically though, while hsa-miR-92a-3p showed a down-regulation in our serum samples with RNA-seq in the discovery cohort, RT-qPCR showed an opposite regulation, and no change in our confirmation cohort. While additional samples may elucidate a resolution, no obvious explanation for these data is currently evident. Lastly, hsa-miR-21-5p has been linked to axonal regeneration following spinal injury (Strickland *et al.*, 2011; Hu *et al.*, 2013), and protection of neurons against ischaemic death (Buller *et al.*, 2010). Therefore, it is possible that the observed reduction in hsa-miR-21-5p expression in serum may reflect decreased hsa-miR-21-5p in neural cells mediating neuroprotective mechanisms.

Using six of our ncRNA biomarkers in combination as a signature to classify the disease state of our samples, we observed a 93.7% classification accuracy between ALS and non-ALS samples, which cross-validated with a second cohort of samples to 73.9% accuracy. This is significant as it demonstrates that these biomarkers may prove to be useful in diagnosis of the disease, but further work is required to test whether these changes are present before symptom onset, something which has been remarked upon as being crucial, including for example in non-symptomatic people with disease-causing mutations (Turner, 2019). However, we recognize that although the signature model has been tested in a new cohort and been cross-validated to 73.9% accuracy, this model is to an extent constrained, exemplified by a limited ability to classify ALS samples in the confirmation cohort. Indeed, our initial results suggest that slight differences in genetic and environmental background may result in the differences that we see in the dysregulation of our biomarkers between the discovery and confirmation cohort, which ultimately impacts on the ability to classify ALS samples using our model. Additionally, these biomarkers by themselves are not yet sufficient to help with prognosis or phenotypic characterization. As such, further work is required to elucidate and include more ncRNA candidates to increase the power of this analysis, along with inclusion of larger and more diverse cohorts with longitudinal samples, to develop this biomarker signature further. Overall, we have demonstrated that there are changes in expression of our ncRNA in ALS, which can be used in combination to stratify patient samples. This is an important initial step in the analysis of all species of ncRNA in the serum for establishing a biomarker signature for ALS and highlights the potential of this approach for other neurodegenerative diseases.

## Supplementary Material

fcaa053_Supplementary_Data

## Abbreviations

ALS =: amyotrophic lateral sclerosis
ALS-FP =: fast-progressing ALS patients
ALS-SP =: slow-progressing ALS patients
ALSFRS-R =: ALS Functional Rating Scale – Revised
AUC =: area under curve
BioMOx =: Oxford Study for Biomarkers in MND/ALS
CA =: classification accuracy
Cq =: quantitative cycle
DM =: disease mimics
ELISA: enzyme-linked immunosorbent assay
fALS =: familial amyotrophic lateral sclerosis
GA =: galaxy-based analysis
HC =: healthy controls
NC =: neurological control
ncRNA =: non-coding RNA
miRNA =: microRNA
MND =: motor neurone disease
NIV =: non-invasive ventilation
PE =: paired end
PEG =: percutaneous endoscopic gastrostomy
piRNA =: piwi-RNA
QA =: Qiagen-based analysis
RNA-seq =: RNA-sequencing
ROC =: receiver operating characteristic
rRNA =: ribosomal RNA
RT-qPCR =: reverse transcription-quantitative PCR
sALS =: sporadic amyotrophic lateral sclerosis
snoRNA =: small nucleolar RNA
snRNA =: small nuclear RNA
tRNA =: transfer RNA
UMI =: unique molecular indexes
