# Supplementary material for: Identification of a potential non-coding RNA biomarker signature for amyotrophic lateral sclerosis
Source: Brain Commun. 2020 Jun 17;2(1):fcaa053. doi: 10.1093/braincomms/fcaa053 (PMC7329382; doi:10.1093/braincomms/fcaa053)
Supplement: fcaa053_Supplementary_Data [file fcaa053_supplementary_data.docx]

**Supplementary Table 1:** Commercially available Primers for TaqMan Advanced miRNA RT-qPCR assays. Primers for piwi-RNA and tRNA fragments were custom designed by Applied Biosystems.

| **MicroRNA Target** | **TaqMan Assay ID** |
| --- | --- |
| hsa-miR-16-5p | 477860_mir |
| hsa-miR-21-5p | 477975_mir |
| hsa-miR-92a-3p | 477975_mir |
| hsa-miR-206 | 477968_mir |
| hsa-miR-718 | 479164_mir |

**Supplementary Table 2:** Reads for the RNA-seq analysis for both QA and GA. The total number of sequenced reads from the MiSeq machine were then reduced to the total number of reads following removal of short reads, adaptor only and in the case of QA, UMI defective reads. Total number of aligned reads that matched to a transcript or genome sequence in the respective analysis and the percentage of total processed reads aligned are then presented (QA includes longer rRNA transcript that GA does not).

| **QA Mappings** | **Healthy Control Pool 1** | **Healthy Control Pool 2** | **Healthy Control Pool 3** | **ALS-SP Pool 1** | **ALS-SP Pool 2** | **ALS-SP Pool 3** | **ALS-FP Pool 1** | **ALS-FP Pool 2** | **ALS-FP Pool 3** | **Overall** |
| --- | --- | --- | --- | --- | --- | --- | --- | --- | --- | --- |
| **Total Number of Sequenced Reads** | 2,760,000 | 2,956,278 | 2,730,436 | 4,355,147 | 5,233,659 | 4,929,195 | 2,750,931 | 2,553,436 | 2,677,749 | 30,946,831 |
| **Total Processed Reads** | 1,573,000 | 1,349,930 | 1,371,117 | 1,302,883 | 1,845,978 | 1,510,358 | 640,452 | 938,296 | 1,496,540 | 12,028,554 |
| **Aligned Reads** | 150,257 | 182,443 | 195,997 | 256,889 | 420,647 | 285,821 | 88,869 | 144,642 | 374,778 | 2,100,343 |
| **% Aligned** | 9.55 | 13.51 | 14.29 | 19.72 | 22.79 | 18.92 | 13.88 | 15.42 | 25.04 | 17.46 |

| **GA Mappings** | **Healthy Control Pool 1** | **Healthy Control Pool 2** | **Healthy Control Pool 3** | **ALS-SP Pool 1** | **ALS-SP Pool 2** | **ALS-SP Pool 3** | **ALS-FP Pool 1** | **ALS-FP Pool 2** | **ALS-FP Pool 3** | **Overall** |
| --- | --- | --- | --- | --- | --- | --- | --- | --- | --- | --- |
| **Total Number of Sequenced Reads** | 2,760,000 | 2,956,278 | 2,730,436 | 4,355,147 | 5,233,659 | 4,929,195 | 2,750,931 | 2,553,436 | 2,677,749 | 30,946,831 |
| **Total Processed Reads** | 1,536,597 | 1,699,714 | 1,499,953 | 1,818,419 | 1,293,036 | 1,506,873 | 756,055 | 1,049,287 | 1,586,095 | 12,746,029 |
| **Aligned Reads** | 90,481 | 79,138 | 120,288 | 147,212 | 74,021 | 97,530 | 33,839 | 56,677 | 120,292 | 819,478 |
| **% Aligned** | 5.89 | 4.66 | 8.02 | 8.10 | 5.72 | 6.47 | 4.48 | 5.4 | 7.58 | 6.43 |

**Supplementary Table 3:** Alignment outputs for QA and GA for the RNA-seq per sample.

| **QA Mappings** | **Healthy Control Pool 1** | **Healthy Control Pool 2** | **Healthy Control Pool 3** | **ALS-SP Pool 1** | **ALS-SP Pool 2** | **ALS-SP Pool 3** | **ALS-FP Pool 1** | **ALS-FP Pool 2** | **ALS-FP Pool 3** |
| --- | --- | --- | --- | --- | --- | --- | --- | --- | --- |
| **miRNA** | 81,006 | 83,137 | 92,971 | 127,907 | 200,603 | 148,876 | 49,829 | 66,034 | 56,410 |
| **Hairpin** | 89 | 481 | 346 | 43 | 113 | 76 | 121 | 155 | 3,109 |
| **piRNA** | 6,329 | 10,385 | 12,530 | 10,299 | 24,975 | 12,974 | 2,913 | 5,224 | 6,911 |
| **rRNA** | 46,939 | 64,109 | 54,988 | 98,046 | 154,999 | 99,893 | 27,050 | 59,578 | 277,426 |
| **tRNA** | 9,962 | 16,724 | 27,004 | 11,369 | 28,760 | 14,503 | 4,557 | 7,115 | 12,444 |
| **mRNA** | 4,119 | 4,786 | 4,837 | 7,227 | 8,788 | 7,505 | 3,506 | 3,332 | 4,519 |
| **Other RNA** | 1,813 | 2,821 | 3,321 | 1,998 | 2,409 | 1,994 | 893 | 3,204 | 13,959 |

**Supplementary Table 3 (continued)**

| **GA Mappings** | **Healthy Control Pool 1** | **Healthy Control Pool 2** | **Healthy Control Pool 3** | **ALS-SP Pool 1** | **ALS-SP Pool 2** | **ALS-SP Pool 3** | **ALS-FP Pool 1** | **ALS-FP Pool 2** | **ALS-FP Pool 3** |
| --- | --- | --- | --- | --- | --- | --- | --- | --- | --- |
| **piRNA** | 17,854 | 14,612 | 15,085 | 17,217 | 15,085 | 24,045 | 21,418 | 11,989 | 12,710 |
| **rRNA** | 9,549 | 6,054 | 5,858 | 5,046 | 4,628 | 33,070 | 750 | 4,511 | 3,432 |
| **snoRNA** | 2,602 | 6,267 | 488 | 2,399 | 4,528 | 2,544 | 265 | 810 | 771 |
| **non_coding** | 111 | 404 | 83 | 442 | 463 | 6,233 | 23 | 32 | 17 |
| **scRNA** | 1 | 14 | 2 | 42 | 42 | 37 | 16 | 3 | 2 |
| **sRNA** | 1 | 5 | 10 | 0 | 0 | 8 | 0 | 3 | 3 |
| **ribozyme** | 38 | 141 | 46 | 0 | 47 | 291 | 28 | 19 | 16 |
| **tRNA** | 36,398 | 47,494 | 32,035 | 41,812 | 38,176 | 60,274 | 20,275 | 14,311 | 14,434 |
| **miRNA** | 16,287 | 15,673 | 23,678 | 14,508 | 16,635 | 3,478 | 45,046 | 25,092 | 28,729 |
| **Mt_rRNA** | 806 | 4,032 | 98 | 952 | 1,306 | 10,143 | 178 | 899 | 221 |
| **Mt_tRNA** | 309 | 283 | 272 | 272 | 437 | 396 | 389 | 373 | 273 |
| **snRNA** | 1,098 | 978 | 571 | 1,822 | 1,830 | 5,380 | 386 | 460 | 315 |
| **scaRNA** | 13 | 36 | 33 | 41 | 27 | 29 | 44 | 51 | 22 |
| **vaultRNA** | 0 | 0 | 4 | 0 | 0 | 0 | 0 | 0 | 16 |

**Supplementary Table 4:** Calculated fold changes of the seven identified biomarkers in RNA-seq and RT-qPCR. For RNA-seq, the analysis in which significance was detected for the biomarker is listed in the first column. Significance of changes for the RT-qPCR confirmation studies are presented in the text and Figure 3A but are summarised here. No fold changes can be presented for MIR206 in the RT-qPCR studies due to a lack of detection in the healthy control samples. * p <0.05, ** p <0.01, *** p < 0.001

|  | **RNA-seq** | | | | **RT-qPCR** | | | | | |
| --- | --- | --- | --- | --- | --- | --- | --- | --- | --- | --- |
|  | **QA/GA?** | **to Healthy Controls** | | **to ALS-SP** | **to Healthy Controls** | | | **to Disease Mimics** | | **to ALS-SP** |
|  |  | **ALS-SP** | **ALS-FP** | **ALS-FP** | **Disease Mimics** | **ALS-SP** | **ALS-FP** | **ALS-SP** | **ALS-FP** | **ALS-FP** |
| **hsa-miR-16-5p** | QA | 1.30 | -3.24 ** | -4.66 *** | -1.37 | -1.63 | -3.66 | -1.19 | - 2.67 *** | -2.45 * |
| **hsa-miR-21-5p** | QA | 1.13 | -3.05 ** | -3.62 *** | -1.35 | -2.00 * | -2.15 * | -1.48 | -1.59 | -1.07 |
| **hsa-miR-92a-3p** | QA | -1.51 | -3.06 ** | -2.11 * | 3.37 * | -1.05 | 1.69 | -3.53 *** | -1.99 ** | 1.77 ** |
| **hsa-miR-206** | GA | 3.91 ** | 2.61 * | -1.30 | N/A | | | | | |
| **hsa-piR-33151** | QA | -4.37 *** | 3.57 ** | 16.68 *** | 4.99 | 1.39 | 2.43 | -3.60 *** | -2.05 * | 1.75 *** |
| **TRV-AAC4-1.1** | GA | -0.04 | 4.48 * | 4.52 * | 3.00 * | 1.27 | 1.86 | -2.36 | -1.61 | 1.46 |
| **TRA-AGC6-1.1** | GA | -2.98 ** | 0.91 | 3.88 *** | 1.60 | -2.75 *** | -1.00 | -4.40 *** | -1.61 | 2.74 |


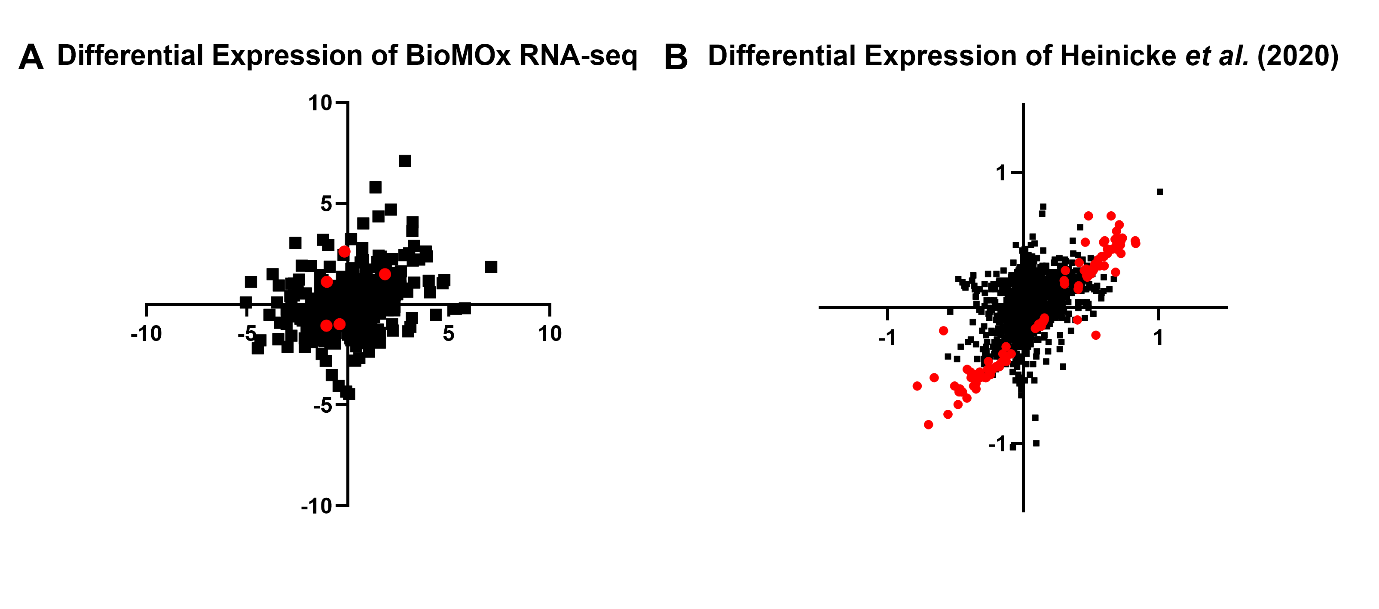


Supplementary Figure 1**:** Correlation of differential expression determined by RNA-seq using the Qiagen-based (QA) and Galaxy-based (GA) analysis. Comparison shown between healthy control and slow- and fast-progressing ALS samples from the BioMOx cohort (**A**) and between healthy control and rheumatoid arthritis patients from Heinicke et al. (2020) (**B**). The Heinicke et al. (2020) dataset was generated also using the QIASeq miRNA NGS kit using 200 ng total RNA from CD8+ T-cells with an alignment of 83.5% and 59.5% of the reads to the genome and our transcriptome using QA and GA respectively. All ncRNA (miRNA and piRNA) picked up by both analyses and differential expression calculated were included in the analysis. Very similar correlation strengths were observed between the two, but the spread of the data differed. Red circles represent ncRNA that showed significant differential expression.


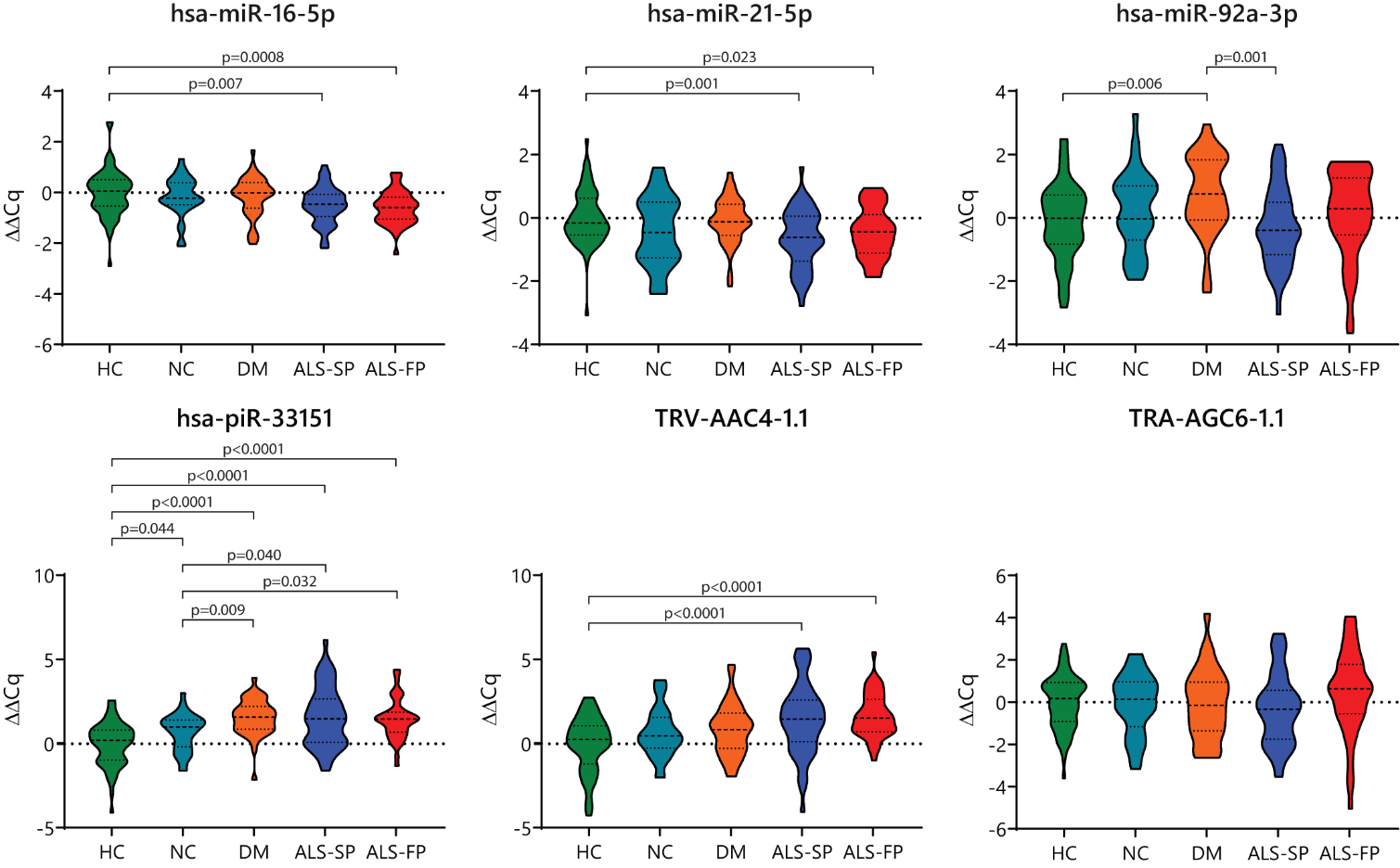


Supplementary Figure 2**:** Differential expression of ncRNA biomarkers in ALS patient serum samples using RT-qPCR in the combined BioMOx discovery and ALS Biomarker Study & Ulm Neurological Biobank confirmation cohorts. Hsa-miR-16-5p/hsa-miR-21-5p/hsa-miR-92a-3p/TRV-AAC4-1.1/TRA-AGC6-1.1: One-way ANOVA with Tukey post-hoc, hsa-piR-33151: One-way ANOVA with Gomes-Howell post-hoc. Normalised to hsa-miR-718 and hsa-piR-31068. Relative expression to the average expression of healthy controls. Healthy control (HC): n=67; neurological controls (NC): n=33; disease mimics (DM): n=43; slow-progressing ALS (ALS-SP): n=50; fast-progressing ALS (ALS-FP): n=45. Violin plot: dotted lines denote top to bottom: 75% quartile, median, 25% quartile.
